# Interaction of Long Working Hours and Sense of Coherence on Objective Total Sleep Time: Cross-Sectional Study From the SLEPT Study

**DOI:** 10.2196/73784

**Published:** 2025-09-03

**Authors:** Kei Muroi, Emi Morita, Sumire Matsumoto, Asuka Ishihara, Sumi Hasegawa, Mami Ishitsuka, Daisuke Hori, Shorato Doki, Tsukasa Takahashi, Shin-ichiro Sasahara, Takashi Kanbayashi, Masashi Yanagisawa, Makoto Satoh, Ichiyo Matsuzaki

**Affiliations:** 1 International Institute for Integrative Sleep Medicine (WPI-IIIS), Tsukuba Institute for Advanced Research (TIAR), University of Tsukuba, Tsukuba, Japan; 2 Forestry and Forest Products Research Institute, Forest Research and Management Organization, Tsukuba, Japan; 3 Graduate School of Comprehensive Human Sciences, University of Tsukuba, Tsukuba, Japan; 4 School of Nursing at Narita, International University of Health and Welfare, Narita, Japan; 5 Institute of Medicine, University of Tsukuba, 1-1-1 Tennodai, Ibaraki, Tsukuba, 305-8575, Japan, 81 029-853-6025; 6 Ibaraki Prefectural Medical Center of Psychiatry, Kasama, Ibaraki, Japan; 7 Department of Molecular Genetics, University of Texas Southwestern Medical Center, Dallas, TX, United States

**Keywords:** actigraphy, long working hours, sleep duration, salutogenesis, worker, employee

## Abstract

**Background:**

Long working hours are a significant risk factor for reduced sleep duration among workers. Sense of coherence (SOC), a dispositional orientation that enhances resilience to daily stressors, may serve as a protective factor for sleep duration under work-related stress. However, previous studies examining SOC and sleep duration have relied on subjective measures, which may be subject to recall bias. The interaction between long working hours and SOC on objective sleep duration has not been previously investigated.

**Objective:**

This study aimed to investigate the interaction between SOC and long working hours on objectively measured total sleep time (TST) among Japanese workers. We hypothesized that individuals with higher SOC would demonstrate less susceptibility to sleep reduction associated with long working hours compared to those with lower SOC.

**Methods:**

A cross-sectional survey was conducted from 2016 to 2017 as part of the Sleep Epidemiology Project at the University of Tsukuba (SLEPT) study among workers in Japan. The 13-item Sense of Coherence scale (SOC-13) was administered to assess participants’ SOC levels, and weekly working hours were self-reported. TST was objectively measured using actigraphy devices worn continuously for 1 week. Long working hours were defined as ≥50 hours per week. Multiple regression analysis was performed with TST as the dependent variable, including long working hours, SOC-13, and their interaction term as independent variables. Simple slope analysis was conducted to examine the interaction effect at different SOC levels of ±1 SD from the mean.

**Results:**

A total of 540 workers were included in the final analysis. The study population had a mean age of 43.2 years, with 41.1% female participants. Mean TST was 322.0 (SD 58.0) minutes, and mean SOC score was 58.3 (SD 11.9). Long working hours were reported by 304 (56.3%) participants. Multiple regression analysis revealed a significant main effect of long working hours on reduced TST (β=−.115, *P*=.023), with workers in the long hours group sleeping 13.5 minutes less per night. Importantly, a significant interaction between long working hours and SOC was observed (β=.147, *P*=.026), indicating that SOC moderated the relationship between long working hours and sleep duration. Simple slope analysis demonstrated that at low SOC levels (−1 SD), long working hours were significantly associated with reduced TST (β=−24.7, *P*=.0015).

**Conclusions:**

Workers with lower SOC experienced significantly greater sleep reduction when working long hours, while those with higher SOC maintained relatively stable sleep duration despite extended work schedules. These findings suggest that interventions aimed at enhancing SOC may be effective in protecting workers’ sleep health, particularly for those unable to reduce their working hours.

## Introduction

Sleep duration is an important biomarker in human sleep, and short sleep duration is associated with increased cardiovascular disease, diabetes, mental disorders, and all-cause mortality [1-3]. In addition, short sleep duration is a risk for occupational accidents, incidents, and presenteeism among workers [4,5]. Japan has the lowest average sleep duration among high-income countries [6], especially among the working-age population. Ikeda et al [7] report that permanent daytime workers in Japan sleep about 360 minutes on average. It is important to identify factors that protect sleep duration in this population.

In workers, long working hours are a risk factor for insufficient sleep [8,9]. International organizations and countries have established standards for maximum working hours. For example, International Labor Organization conventions stipulate a maximum of 48 hours of work per week [10]. In Japan, the Labor Standards Law stipulates that an employer shall not make an employee work more than 8 hours per day or 40 hours per week [11]. An agreement should be concluded between an employer and an employee and submitted to the Labor Standards Inspection Office when the employer wants to extend working hours or have an employee work on holidays. As a general rule, the upper limit of overtime work is 45 hours per month or 360 hours per year. When there are special temporary circumstances, the upper limit can be up to 720 hours per year or less than 100 hours per month or up to an average of 80 hours per month over multiple months [11]. Assuming 45 hours of overtime per month, this translates into an approximate 50-hour workweek, which is roughly in line with the conventions set forth by the International Labor Organization. Against this background, long working hours are often defined as around 50 hours per week, and a meta-analysis by Wong et al [9] reported that working hours in excess of around 50 hours per week are associated with risk of cardiovascular and cerebrovascular diseases, depression, anxiety disorder, and sleep disorder in workers. They also suggest that the mechanism of short sleep duration due to long working hours is related to high work stress and the sympathetic nervous system [12,13], in addition to difficulty in securing sleep. Research on the effect of increased sleep on reduced work hours is limited, although a study by Schiller et al [14] reported that a 25% reduction in weekly work hours resulted in an increase of 23 minutes of sleep. On the other hand, it has been noted that sleep duration may not increase [15]; therefore, methods other than the reduction of working hours are important to maintain workers’ sleep duration.

Salutogenesis is an approach to human health that examines the factors that promote and maintain physical and mental health [16]. Antonovsky defined sense of coherence (SOC) as the ability to maintain health in the face of stressful events and situations [17,18]. SOC is defined as comprehensibility, manageability, and meaningfulness [17,18]. People with high SOC have a higher ability to cope with stress and maintain physical and mental health in the face of stress, and associations between SOC and depression and all-cause mortality have been reported [19,20]. Furthermore, SOC has been reported to protect against stress in workers and even against serious stresses such as post-traumatic stress disorder [21,22]. Long working hours are a stressful situation, and workers’ SOC may be associated with sleep duration.

To our knowledge, only 2 studies have previously examined the association between SOC and sleep duration: in a study of software office workers, Morita et al [23] reported that workers with high SOC maintained more than 6 hours of sleep per day as compared with workers with low SOC; in a study of civil servants, Kósa et al [24] also reported a U-shaped association between SOC and sleep duration, with a positive association between SOC and sleep duration of up to 8 hours per day. However, the interviews in these studies focused only on subjective sleep duration, which may be subject to recall bias [25]. Since subjective sleep duration is reported to be longer than actual sleep duration, it is more important to measure objective sleep duration. The gold standard for objective sleep duration is polysomnography (PSG) [26], but it is not suitable for measuring large populations. Recently, actigraphy, with its built-in accelerometer, has been used in epidemiologic studies to measure objective sleep duration, with a reported agreement rate of approximately 90% with PSG [27,28]. Furthermore, previous studies have not taken into account the factor of working hours in the relationship between sleep duration and SOC, and it would be useful to examine the interaction between long working hours and SOC, as SOC can help maintain health under stress. It is important to examine interactions in epidemiology to consider effective interventions to improve outcomes with limited resources [29]. By examining interactions, we can gain clues about the reasons and mechanisms whereby exposure factors affect outcomes [30]. While the interaction between SOC and long working hours in workers’ psychological stress has been studied [21], the interaction between SOC and long working hours with sleep duration has not been reported. Examining the interaction between long working hours and SOC in objective sleep can provide important insights into the protection of workers’ sleep.

Hence, we examined the interaction between long working hours and SOC by using actigraphy to measure sleep duration in workers.

## Methods

### Study Design and Recruitment

#### Study Framework and Timeline

Data for this study were obtained from the Sleep Epidemiology Project at University of Tsukuba (SLEPT) study, a comprehensive sleep epidemiology investigation conducted jointly by the University of Tsukuba and the International Institute for Integrative Sleep Medicine, University of Tsukuba. The SLEPT study uses a cross-sectional design with prospective data collection conducted from 2016 to 2017. Data were collected through multiple complementary methods: self-administered questionnaires, participant sleep diaries maintained for 7 consecutive days, and objective actigraphy measurements.

#### Study Settings and Participant Recruitment

The study was conducted across 4 diverse workplace settings in Japan: a national university hospital in Ibaraki Prefecture, a national research institute, a corporate research institute, and a medical corporation in Tokyo. To maximize participation across diverse worker populations, a multi-modal recruitment approach was implemented during the recruitment period from 2016 to 2017. The recruitment strategy encompassed multiple complementary approaches. The participants were recruited by use of flyers, posters, workplace group emails, and workplace web-based bulletin boards. Some participants were referred by research staff or other study participants.

During the systematic recruitment process conducted across all 4 sites, potential participants received comprehensive study information including objectives, time commitments, data collection procedures, and the voluntary nature of participation. Interested individuals were scheduled for enrollment sessions where informed consent was obtained and study materials were distributed.

#### Participation Incentives and Feedback

Although no monetary compensation was provided, several non-monetary incentives were offered to encourage participation and provide value to workers. All participants who completed the study protocol received personalized sleep reports offering insights into their individual sleep patterns and characteristics. Additionally, basic sleep hygiene information and educational materials were provided to enhance participants’ understanding of healthy sleep practices.

### Measurements

#### Basic Attributes

Age, sex, height, weight, drinking habits, exercise habits, and smoking status were surveyed. Survey items related to exercise habits were “Yes” (=“I exercise at least 30 min, twice a week, for 1 y”) or “No” (=“other”) [31]; those related to drinking habits were “Yes” (=“more than once a week”) or “No” (=“less than once a week”); and those related to smoking habits were “never smoker,” “former smoker,” or “current smoker.” They were also asked about any diseases the participant was currently being treated for, and any medications the participant was currently taking were also surveyed.

#### Occupational Factors

The participants were asked about their working hours (hours) for the last week, their daily 1-way commuting time (minutes), and their employment status as defined by the Ministry of Health, Labor and Welfare (full-time regular employees or regular staff, full-time employees with short hours, part-time employees, contract employees, managers or staff, and others). They were also asked about whether they were currently engaged in shift work.

The Brief Scales for Job Stress (BSJS) [32] was used for job stress; the BSJS is a 20-item questionnaire based on a job demands-control-support model similar to the Job Content Questionnaire [33]. All participants were asked, “Please select the statement about your current work environment that most closely matches your feelings.” Responses were rated on a 4-point scale (1=“disagree” to 4=“agree”) and mean scores (range: 1.00‐4.00) were calculated for 6 subscales: “workload,” “mental workload,” “interpersonal relationships,” “job control,” “reward from work,” and “support from colleagues and superiors.” The reliability and validity of this scale have been reported [34-36]. Workload, mental workload, and interpersonal relationship were defined as stress factors, and job control, reward from work, and support from colleagues and superiors as stress-buffering factors. The Cronbach α coefficient for the BSJS in this study was 0.766.

#### Objective Total Sleep Time

The MTN-220 (ACOS Co Ltd.) was used to record total sleep time (TST). Participants wore the MTN-220 on their trunk by clipping it to their trousers or pants, 24 hours a day for 1 week, except when bathing; the MTN-220 records activity using a built-in 3-axis acceleration sensor [37]. In a laboratory validation study, the agreement with PSG data ranged from 84.7% to 85.4% [37]. Data were extracted from the MTN-220 device by use of SleepSignAct2 software (Kissei Comtec Co. Ltd.). Sleep and wake detection from MTN-220 data followed the previously reported algorithm of SleepSignAct2 [37]. A sleep diary was used to measure subjective sleep patterns. Participants were required to estimate their sleep duration each morning and record the results in a sleep diary. After data acquisition, the data from the sleep measurements and the sleep diary were checked for consistency. Objective sleep duration on actigraphy was calculated after determining the wake times and bedtimes in the participants’ sleep diaries, and the TST (minutes) was the average of all nights of data collected by actigraphy.

#### Sense of Coherence

The 13-item version of the Sense of Coherence scale (SOC-13) was used to assess SOC [18]. A shortened version of the original 29-item SOC scale, the SOC-13 scale was developed by Antonovsky [18,38]. The SOC-13 includes items categorized into 3 subscales: Comprehensibility (consisting of 5 items: 2, 6, 8, 9, and 11), Manageability (consisting of 4 items: 3, 5, 10, and 13), and Meaningfulness (consisting of 4 items: 1, 4, 7, and 12). The final score for each participant is the sum of the scores of the items, including reversed scores for questions 1, 2, 3, 7, and 10 (where a score of 7 is considered as 1, 6 as 2, 5 as 3, 4 as 4, 3 as 5, 2 as 6, and 1 as 7). Scores range from 13.0 to 91.0 points, with higher total scores indicating a stronger SOC. The Japanese version of the SOC-13 questionnaire [39], which has been translated and validated in this language, was used in our study. Members of the Society for Theory and Research on Salutogenesis are authorized to use this questionnaire for academic research [40]. The authors, KM, SD, and SS, are members of the Society for Theory and Research on Salutogenesis. The Cronbach α coefficient for the SOC-13 in this study was 0.856.

### Statistical Analysis

A total of 785 workers participated in the study. Written informed consent was obtained from all the participants. In total, 4 participants subsequently withdrew their consent, and their data were deleted. An additional 36 participants were excluded because they did not meet the eligibility criteria. This was due to inconsistencies between the sleep measurement data and the sleep diaries. Of the remaining 745 participants, 205 participants having sleep disorders such as sleep apnea, having a history of psychiatric disorders, taking sleeping pills or psychotropic drugs, working short hours or part-time, or missing data were excluded, leaving a total of 540 participants for analysis.

Long working hours were defined as more than 50 hours of work per week. BMI was calculated from height and weight and divided into 3 groups (underweight:<18.5, normal weight: 18.5‐24.9, overweight:≥25), age was divided into 5 groups (20‐29, 30‐39, 40‐49, 50‐59, and 60 or older), and commuting time was doubled to daily 1-way commuting time. Multiple regression analysis was conducted with TST as the dependent variable and with long working hours, SOC-13, and the interaction term between the 2 variables as the independent variables. The SOC-13 was centralized to avoid multicollinearity due to the correlation between the main effect term and the interaction term. Age, sex, BMI, drinking habits, exercise habits, smoking status, BSJS, presence of shift work, and commuting time were entered as confounders in the multiple regression model. When an interaction was observed, we performed simple slope analysis and plotted the results based on the studies by Aiken and West [41] and Cohen et al [42] and tested the simple slopes of the regression lines corresponding to combinations of low (one standard deviation below the mean: −1SD) and high (one SD above the mean:+1 SD) levels of the SOC. Furthermore, we made the plot of the 2-way interaction between long working hours and SOC. The statistical software used was R (version 4.3.1; R Foundation for Statistical Computing), the significance level was 0.05 on both sides, and the pequod package [43] was used for single slope analysis.

### Ethical Considerations

#### Ethics Review and Approvals

This study was conducted in accordance with the ethical standards of the Declaration of Helsinki and was approved by the Medical Ethics Committee of the University of Tsukuba (approval number: 1065‐10). The research protocol underwent comprehensive review to ensure compliance with national research ethics guidelines and institutional policies for human subjects research. All procedures involving human participants were conducted in accordance with the ethical standards of the institutional or national research committee and with the 1964 Helsinki Declaration and its later amendments or comparable ethical standards.

#### Informed Consent

Written informed consent was obtained from all participants prior to their enrollment in the study. Participants were provided with comprehensive information about the study objectives, procedures, potential risks and benefits, data collection methods, and their rights as research participants. The consent process included detailed explanations of the actigraphy measurement procedures, questionnaire requirements, and sleep diary maintenance. Participants were explicitly informed of their right to withdraw from the study at any time without penalty or impact on their employment or health care. Four participants subsequently exercised their right to withdraw consent, and their data were immediately deleted from all study databases and analyses in accordance with their wishes and ethical requirements.

#### Privacy and Confidentiality Protection

All study data were deidentified and anonymized to protect participant privacy and confidentiality. Personal identifiers were separated from research data and stored securely with access restricted to authorized research personnel only. Study databases were password-protected and maintained on secure servers with appropriate data encryption protocols. Participants were assigned unique study identification numbers to ensure anonymity throughout the data collection, analysis, and reporting processes. No individual participants can be identified from any published results, tables, figures, or supplementary materials. Data-sharing procedures adhere to institutional policies and ethical requirements for protection of participant privacy.

#### Compensation and Incentives

No monetary compensation was provided to study participants. However, as an incentive for participation and to provide value to participants, individual sleep measurement results and basic sleep health information were provided as feedback to all participants who completed the study protocols. This feedback was presented in a summary format to help participants better understand their sleep patterns while maintaining the scientific integrity of the study. The feedback process was designed to be educational rather than diagnostic, with appropriate disclaimers about the research nature of the measurements.

#### Participant Identification and Images

This manuscript and all supplementary materials contain no images, photographs, or other materials that could potentially identify individual study participants. All data presentations use aggregated statistical summaries, and no individual-level data points are displayed in ways that could compromise participant anonymity. The research team ensured that all tables, figures, and data visualizations maintain participant confidentiality and comply with privacy protection requirements.

## Results

Table 1 shows the descriptive characteristics of the participants. The mean overall sleep duration was 322 (SD 58.0) minutes, and the mean SOC was 58.3 (SD 11.9). The long hours group (*≥*50 hours/week) showed a shorter mean TST (<50 hours/week: mean 332 SD 56.1 minutes; ≥50 hours/week: mean 315, SD 58.5 minutes; *P*<.001); no difference was found between the 2 groups for SOC (<50 hours/week: mean 58.0, SD 11.8; ≥50 hours/week: mean 58.5, SD 12.0; *P*=.62).

**Table 1.** Demographic and occupational characteristics of Japanese workers by weekly working hours in a cross-sectional analysis from the SLEPT^a^ study (N=540). Cross-sectional analysis from the SLEPT study conducted in 4 workplaces in Ibaraki and Tokyo prefectures, Japan (2016-2017). Participants were full-time daytime workers aged 20-60 years. Working hours were categorized as <50 hours/week (n=236, 43.7%) and ≥50 hours/week (n=304, 56.3%).

	Working hours		*P* value
	<50 hours/week (n=236)	≥50 hours/week (n=304)	
Sex, n (%)			
Male	85 (36.2)	180 (59.2)	<.001^b^
Female	150 (63.8)	124 (40.8)	
Age (years), n (%)			
20‐29	35 (14.8)	54 (17.8)	.12^b^
30‐39	70 (29.7)	82 (27)	
40‐49	68 (28.8)	73 (24)	
50‐59	46 (19.5)	82 (27)	
60 and older	17 (7.2)	13 (4.3)	
BMI, n (%)			
Underweight	23 (9.7)	18 (5.9)	.042^b^
Normal weight	166 (70.3)	242 (79.6)	
Overweight	47 (19.9)	44 (14.5)	
Drinking habit, n (%)			
No	81 (34.3)	69 (22.7)	.004
Yes	155 (65.7)	235 (77.3)	
Exercise habit, n (%)			
No	188 (79.7)	244 (80.3)	.91
Yes	48 (20.3)	60 (19.7)	
Smoking status, n (%)			
Never smoker	180 (76.3)	229 (75.3)	.94
Current smoker	20 (8.5)	25 (8.2)	
Former smoker	36 (15.3)	50 (16.4)	
Shift work, n (%)			
No	219 (92.8)	262 (86.2)	.018
Yes	17 (7.2)	42 (13.8)	
BSJS^c^, mean (SD)			
Workload	1.83 (0.630)	2.60 (0.740)	<.001^d^
Mental workload	1.95 (0.700)	2.46 (0.710)	<.001^d^
Interpersonal relationship	1.75 (0.670)	2.02 (0.78)	<.001^d^
Reward from work	2.81 (0.820)	3.01 (0.77)	.004^d^
Support from colleagues and superiors	3.00 (0.650)	2.90 (0.630)	.072^d^
Job control	2.83 (0.720)	2.88 (0.770)	.500^d^
Commuting time	73.4 (75.5)	62.9 (50.2)	.053^d^
SOC-13^e^	58.0 (11.8)	58.5 (12.0)	.62^d^
TST^f^	332 (56.1)	315 (58.5)	<.001^d^

a SLEPT: Sleep Epidemiology Project at the University of Tsukuba.

b Chi-square test.

c BSJS, Brief Scales for Job Stress.

d Unpaired *t* test.

e SOC-13: 13-item Sense of Coherence scale.

f TST: total sleep time.

Table 2 shows the results of the multiple regression analysis with TST as the dependent variable and long hours worked, SOC-13, and the interaction of the 2 variables as the independent variables. A main effect of long working hours was found (β=−.115, *P*=.023), as well as an interaction between long working hours and SOC (β=.147, *P*=.026), while SOC alone was not associated with TST (β=−.0537, *P*=.45).

Table 3 shows the results for the covariates in the multiple regression analysis. Female sex (β=0.155, *P*=.0020), having an overweight BMI (β=−0.0954, *P*=.030), and working a shift (β=−0.0914, *P*=.044) were associated with TST. The adjusted *R*-squared value on multiple regression analysis was 0.0560.

Table 4 shows the simple slope analysis for moderating effects at (−1 SD) low and (+1 SD) high levels of SOC between TST and long working hours. Only low levels of SOC were significant. At low levels of SOC, long working hours significantly and negatively predicted TST (β=−24.7, *t*_516_=−3.19, *P*=.0015). However, at high levels of SOC, long working hours did not significantly predict TST (β=−2.21, *t*_516_=−0.29, *P*=.78).

**Figure 1.**
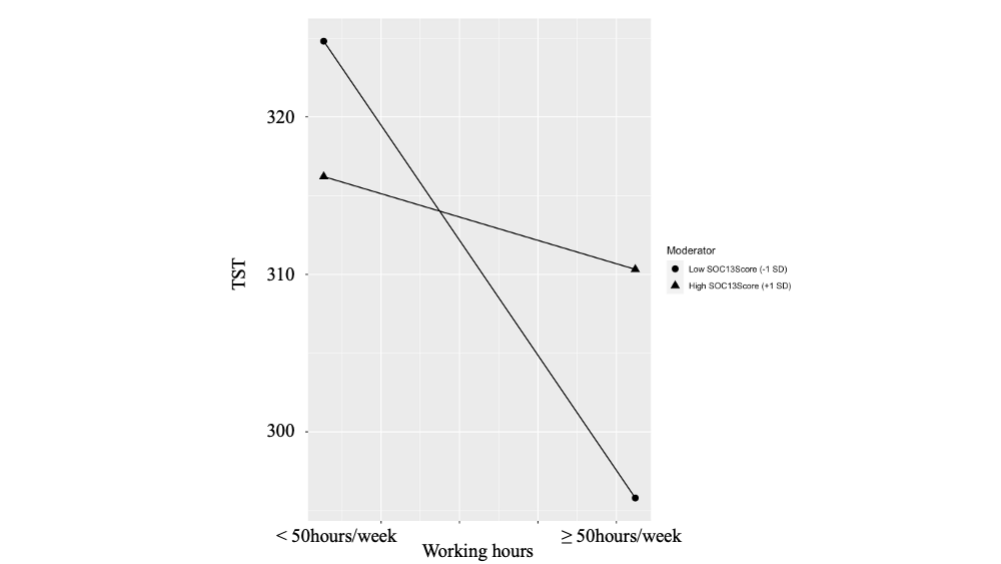
Cross-sectional analysis from the Sleep Epidemiology Project at University of Tsukuba (SLEPT) study conducted across 4 workplaces in Ibaraki and Tokyo prefectures, Japan (2016‐2017). The plot illustrates the significant 2-way interaction (β=0.147, *P*=.026) between working hours (categorized as <50 vs ≥50 hours/week) and sense of coherence on total sleep time measured by 7-day waist-worn actigraphy. Low sense of coherence (SOC) represents scores 1 SD below the mean (–1 SD; triangle markers, approximately 46.4 points), while high SOC represents scores 1 SD above the mean (+1 SD; circle markers, approximately 70.2 points). Workers with low SOC showed a significant 24.7-minute reduction in sleep duration when working ≥50 hours/week (*P*=.0015), whereas workers with high SOC showed no significant sleep reduction (2.21 minutes, *P*=.78). The interaction demonstrates that a sense of coherence protects against sleep loss associated with long working hours. The y-axis shows total sleep time in minutes; the x-axis shows working hour categories. SOC-13: sense of coherence measured by 13-item scale; TST: total sleep time.

Figure 1 shows the plot of the 2-way interaction between long working hours and SOC on TST.

**Table 2.** Multiple regression analysis results examining the interaction between long working hours and sense of coherence on objectively measured total sleep time among Japanese workers (N=540). Results from the cross-sectional SLEPT^a^ study of Japanese workers across 4 workplaces (2016-2017). Dependent variable: 7-day actigraphy-measured total sleep time. The model is adjusted for demographic, lifestyle, and occupational factors.

	Β^b^	SE	*t* test (*df*)	β^c^	*P* value
Working hours (Ref^d^ <50 hours/week)					
≥50 hours/week	−13.5	5.89	−2.29 (516)	−.115	.023
SOC-13^e^	−0.261	0.344	−0.760 (516)	−.0537	.45
Working hours≥50 hours/week×SOC-13	0.943	0.423	2.23 (516)	.147	.026

a SLEPT: Sleep Epidemiology Project at the University of Tsukuba.

b Β: nonstandard coefficient.

c β: standard coefficient.

d Ref: reference.

e SOC-13: 13-item Sense of Coherence scale.

**Table 3.** Covariate effects in multiple regression analysis predicting total sleep time among Japanese workers with long working hours interaction model (N=540). Effects of demographic, lifestyle, and occupational covariates in the working hours×SOC^a^ interaction model from the SLEPT^b^ study (Japan, 2016-2017).

	Β^c^	SE	*t* test (*df*)	β^d^	*P* value
Female sex (ref: male)	18.0	5.80	3.11 (516)	.155	.0020
Age (years; ref: 20‐29 years)					
30‐39	−2.43	7.81	−0.311 (516)	−.0188	.76
40‐49	−15.3	8.10	−1.89 (516)	−.116	.059
50‐59	−10.6	8.78	−1.21 (516)	−.0777	.23
60 and older	−19.8	13.0	−1.52 (516)	−.0783	.13
BMI (ref^e^: normal weight)					
Underweight	−0.157	9.47	−0.0165 (516)	−.000720	.99
Overweight	−14.8	6.81	−2.17 (516)	−.0954	.030
Having a drinking habit (ref: not having one)	2.87	5.74	0.500 (516)	.0221	.62
Smoking status (ref: never smoker)					
Current smoker	−0.961	9.24	−0.104 (516)	−.00458	.92
Former smoker	2.18	7.12	0.306 (516)	.0138	.76
Having an exercise habit (ref: not having one)	−0.998	6.34	−0.158 (516)	−.00688	.88
Doing shift work (ref: not doing shift work)	−17.0	8.41	−2.02 (516)	−.0914	.044
BSJS^f^ workload	−3.39	4.29	−0.790 (516)	−.0465	.43
BSJS mental workload	0.774	4.52	0.171 (516)	.0100	.86
BSJS interpersonal relationship	5.60	4.16	1.34 (516)	.0716	.18
BSJS reward from Work	−1.09	3.83	−0.285 (516)	−.0150	.78
BSJS job control	4.28	3.90	1.10 (516)	.0548	.27
BSJS support from colleagues and superiors	2.81	4.71	0.596 (516)	.0310	.55
Commuting time	−0.0323	0.0400	−0.808 (516)	−.0348	.42

a SOC: sense of coherence.

b SLEPT: Sleep Epidemiology Project at the University of Tsukuba.

c Β: nonstandard coefficient.

d β: standard coefficient.

e Ref: reference.

f BSJS: Brief Scales for Job Stress.

**Table 4.** Simple slope analysis examining the effect of long working hours on total sleep time at different levels of sense of coherence among Japanese workers (N=540). Analysis following significant working hours×SOC^a^ interaction in the SLEPT^b^ study (Japan, 2016-2017).

	β^c^	SE	*t* test (*df*)	*P* value
Low SOC-13^d^ (−1 SD)	−24.7	7.76	−3.19 (516)	.0015
High SOC-13 (+1 SD)	−2.21	7.76	−0.29 (516)	.78

a SOC: sense of coherence.

b SLEPT: Sleep Epidemiology Project at the University of Tsukuba.

c β: simple slope.

d SOC-13: 13-item Sense of Coherence scale.

## Discussion

### Principal Findings

To our knowledge, this is the first report examining the association of SOC with objective sleep duration. The TST of the participants was 322 minutes, which was less than the national average. Previously, Takahashi et al [44] measured the sleep duration of 55 Japanese workers by use of actigraphy and reported an average TST of 384 minutes. By comparison, the sleep duration of the present study population was shorter; on the other hand, the mean SOC of the present study population was 58.5, which was comparable to the national survey sample [39]. The reliabilities of the BSJS and SOC-13 in this study were confirmed, as the Cronbach α coefficients exceeded 0.70 [45].

Multiple regression analysis showed that high BMI and shift work were associated with shorter sleep duration, consistent with the results of previous studies [46-48]. Our results showed that women tended to sleep more than men. In a previous study of sex differences in sleep duration among adult non–shift workers in 47 countries, men tended to have shorter sleep durations measured by wearable devices than those of women throughout their lifetime, consistent with our results [49]. On the other hand, sleep duration measured by actigraphy in Japanese was shorter for women than for men, which was speculated to be because women in Japan have more domestic roles [50]. The reason for the difference between our results and those of this previous study may be the method of recruiting participants in the present study. Since women are more health conscious and have more health behaviors than men [51,52], it is possible that the study attracted women who were trying to get more sleep as a result of their health behaviors.

A decrease in TST was observed for long working hours (*≥*50 hours/week) alone, while an interaction between long working hours and SOC was associated with an increase in TST. Single slope analysis showed that both long hours worked groups decreased TST, but that lower SOC was associated with a significantly greater decrease in TST. The interaction results in this study suggest that the effect of long working stress varies with SOC. Recent studies suggest that sleep disturbances are not directly caused by stress but rather by stress coping and reactivity [53,54] and that those with higher SOC are more likely to develop coping behaviors and use more appropriate stress coping strategies, such as seeking assistance, during stress load and seeking help [18]. Since high SOC plays an important role in coping with stress, it is possible that workers’ SOC may be protective in sleep. Furthermore, although studies are limited to health care workers, biological mechanisms have been reported to associate stress with SOC and the dorsolateral prefrontal cortex (DLPFC) [55,56], suggesting that high SOC is associated with a high-functioning DLPFC. With respect to stress-coping mechanisms, the prefrontal cortex plays an important role in translating stress experiences into adaptive behaviors by integrating cognitive and emotional behaviors and promoting neuroendocrine and autonomic nervous system flexibility in response to stress [57,58]. Those with higher SOC have associated higher functioning of the DLPFC and may be better adapted to work stress in long working hours. Further investigation of the biological mechanisms in stress, SOC, and sleep duration is warranted.

### Implications

An approach that focuses on SOC to protect workers’ duration is warranted [14,15], as it has been suggested that reducing working hours alone may not be sufficient to maintain sleep duration. SOC has been suggested to grow in adulthood, and to improve SOC, it is important to increase generalized resistance resources (GRRs), or resources that can be used in coping with stress [59,60]. Within the framework of SOC-GRRs, increasing resources in the workplace is important and can be used as an occupational health measure. Useful GRRs in the workplace include good interpersonal relationships, employee decision-making authority, and opportunities to use skills and knowledge [60]. For occupational health professionals and workplace managers, focusing on GRRs in the workplace and increasing GRRs could help increase workers’ sleep duration in addition to reducing working hours.

### Limitations

This study was a cross-sectional study, so causal relationships are unknown. In addition, there is a generalizability issue because the study population was white-collar workers. Furthermore, there is a possibility of selection bias, which is a healthy worker effect. The multiple regression analysis resulted in an adjusted *R*^2^ of 0.0560, which is not a good fit for the model. It is possible that unmeasured confounders such as marital status and economic status [61,62] were not adjusted for in the present study. In addition, it has been reported that 31% to 55% of sleep duration is related to genetic factors [63,64], and future analyses should take genetic factors into account to construct a model to predict sleep duration. Furthermore, since a study of healthy college students aged 18 to 40 years reported that waist-worn actigraphy devices underestimated TST in comparison with arm-worn devices [65], it is possible that the present waist-worn actigraphy device (ACOS MTN-220) also underestimated TST in the same way. Therefore, electroencephalography-based sleep tracking devices similar to PSG would be suitable for more accurate evaluation of objective sleep measurement. Finally, since the working hours surveyed in this study are a subjective assessment, an objective assessment such as reporting using a timecard or smartphone applications would be more accurate [66].

### Conclusions

The interaction between long working hours (*≥*50 hours/week) and SOC for workers indicated that lower SOC could lead to shorter sleep duration. The results suggest that an SOC-focused approach is important to maintain workers’ sleep duration. Future studies should include large-scale follow-up and investigation of the biological mechanisms.

## Abbreviations

BSJS: Brief Scales for Job Stress
DLPFC: dorsolateral prefrontal cortex
GRR: generalized resistance resource
PSG: polysomnography
SLEPT: Sleep Epidemiology Project at University of Tsukuba
SOC: sense of coherence
SOC-13: 13-item Sense of Coherence scale
TST: total sleep time
